# The causal role of plasma protein ratios in ulcerative colitis: insights from mendelian randomization and single-cell sequencing

**DOI:** 10.1515/med-2026-1434

**Published:** 2026-08-10

**Authors:** Kuo Kang, Linfeng Wei, Xuanxuan Li, Shalong Wang, Changhao Huang, Zhiwei Wu

**Affiliations:** Department of General Surgery, The Second Xiangya Hospital Central South University, Changsha, Hunan, China; Hunan Key Laboratory of Precise Diagnosis and Treatment of Gastrointestinal Tumor, Xiangya Hospital Central South University, Changsha, Hunan, China; Department of Oncology, Xiangya Hospital, Central South University, Changsha, Hunan, China; National Clinical Research Center for Geriatric Disorders, Xiangya Hospital, Central South University, Changsha, Hunan, China; Department of Organ Transplantation, Xiangya Hospital Central South University, Changsha, Hunan, China

**Keywords:** DLL1/TGFBR2 ratio, ulcerative colitis, Mendelian randomization

## Abstract

**Objectives:**

This Mendelian randomization study aimed to systematically investigate the causal associations of 2,821 plasma protein ratios with UC susceptibility and validate potential biomarkers through multi-omics approaches.

**Methods:**

This study employed a two-sample Mendelian randomization (MR) approach using publicly available genetic data from genome-wide association studies (GWAS), with single nucleotide polymorphisms (SNPs) serving as instruments for exposures and outcome variables. Using the two-sample MR approach, we assessed the associations between 2,821 plasma protein ratios and UC and performed a series of sensitivity analyses to enhance the robustness of our findings. Additionally, single-cell sequencing was used to validate and explore the MR results. And ELISA assays were carried to validate the expression of certain protein ratios.

**Results:**

The inverse-variance weighted method identified significant relationships between 165 plasma protein ratios and UC (FDR<0.05). UC susceptibility, per one standard deviation increase in genetically predicted protein ratios, ranged from 0.539 (DCTN1/IKBKG, 95 % CI: 0.408–0.712) to 2.078 (CCT5/DCTN1, 95 % CI: 1.607–2.686). Various sensitivity analyses further confirmed the robustness of these causal relationships. Single-cell sequencing further revealed significant differences in DLL1/TGFBR2 ratios between UC and healthy controls (p<0.0001), with a moderate yet significant elevation in CCT5/DCTN1 ratios (p<0.01) and no notable difference in BCR/DCTN1 ratios. Validation using ELISA in 26 pairs of UC patients and healthy controls showed a significantly elevated DLL1/TGFBR2 ratio in the UC group (p<0.001), while CCT5/DCTN1 and BCR/DCTN1 ratios showed no differences.

**Conclusions:**

The findings reveal a robust causal relationship between the DLL1/TGFBR2 plasma protein ratio and UC, offering novel insights into the diagnosis and treatment of the disease.

## Introduction

Ulcerative colitis (UC) is a chronic inflammatory disease primarily affecting the colonic mucosa, classified as a subtype of inflammatory bowel disease (IBD) [1]. The primary symptoms of UC patients are recurrent bloody stools and diarrhea, which can be life-threatening in severe cases, often requiring long-term or lifelong medication [2]. The pathogenesis of UC is multifaceted, involving genetic alterations, intestinal immune system imbalance, particularly mucosal immune dysregulation, and environmental factors [3]. Epidemiological data indicate that UC can be diagnosed at any age, with peak incidence in young adults (20–49 years in China), showing no significant gender differences. Globally, the incidence and prevalence of UC have steadily risen in parallel with the progression of industrialization [4]. In the past 20 years, the incidence and prevalence of UC have markedly increased in China [5], Approximately 10 % of UC patients are diagnosed before the age of 20 [6], with the disease imposing significant economic burdens on families and society, while also severely impacting patients’ mental health and quality of life [7].

Proteomics plays a critical role in elucidating disease mechanisms and identifying potential therapeutic targets. In previous proteomics research, immunoglobulin G (IgG) and IgA1 have been identified as plasma proteins closely associated with UC disease activity [8]. IgA is also considered a potential glycoprotein target, as its glycosylation may affect FcαR receptor binding and trigger pro-inflammatory or anti-inflammatory responses, serving as a diagnostic biomarker [9].

MR was initially developed as an alternative to randomized controlled trials (RCTs), using genetic instruments instead of the traditional exposure-disease paradigm. According to Mendelian genetics, genetic information is randomly allocated at conception, preceding the onset of any disease manifestations and thereby reducing confounding bias [10]. However, the causal role of plasma protein ratios (versus individual proteins) in UC pathogenesis remains unclear, and no studies have systematically distinguished truly causal biomarkers from secondary inflammatory phenomena. To address these gaps, we conducted the first large-scale Mendelian randomization study analyzing 2,821 plasma protein ratios, followed by single-cell sequencing validation to pinpoint mucosa-specific protein network dysregulation. Crucially, we further evaluated the diagnostic potential of the prioritized DLL1/TGFBR2 ratio through clinical cohort assays, establishing a translational pipeline from genetic inference to biomarker discovery.

## Methods

### Study design

**Figure 1:**
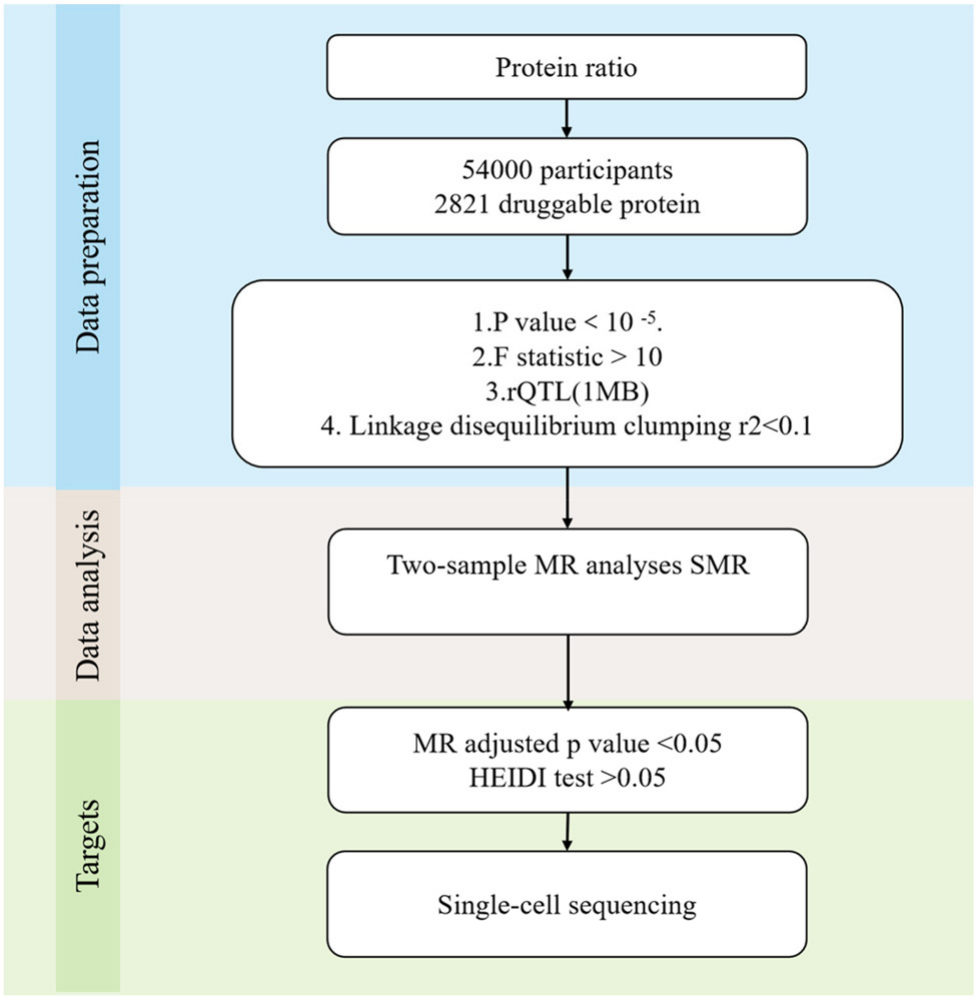
Study design.

This study investigated the causal relationship between 2,821 plasma protein ratios and UC incidence. The MR analysis adhered to three fundamental assumptions: 1, the selected genetic instruments must be strongly associated with the exposure; 2, they must not be influenced by confounding variables; 3, their effect on the outcome must be mediated solely through the specified exposure. Single-cell sequencing data from the GEO database were analyzed to validate and further explore the MR findings. The study design is illustrated in Figure 1.

### Data sources

Genetic data on plasma protein ratios were obtained from a recent GWAS study using proteomics data from 53,116 European-ancestry participants in the UK Biobank (UKB) cohort, following these exclusion criteria: [1] individuals with autoimmune/complex inflammatory diseases; [2] incomplete proteomic profiling; [3] non-European genetic background (determined by principal component analysis). UC outcome data were sourced from the FinnGen study R10 release (n=408,524), which applied the following exclusion protocols: [1] age <18 years; [2] IBD subtype misclassification; [3] duplicate genetic samples. Single-cell data (GSE114374) specifically included two treatment-naive UC patients and two healthy controls. A detailed summary of the data sources for all studied phenotypes (including Population, Data Source, and Website address) is provided in Table S1.

### Instrument selection

To ensure sufficient SNPs for analysis, a significance threshold (p<1×10^−5^) was set. SNPs with weak instrument strength (F statistic <10) were excluded. Independent SNPs meeting the predefined criteria were selected to construct genetic instruments for subsequent statistical analysis.

### Confounding control strategy

Three-level confounding adjustment was implemented: [1] Source-data level: Both UKB and FinnGen GWAS adjusted for age, sex, genotyping array, and genetic principal components; [2] Analysis level: MR-Egger intercept test evaluated directional pleiotropy; [3] Post-hoc level: Bidirectional MR and positive control analyses tested reverse causation.

### ELISA assay

Blood samples were collected from 26 treatment-naive UC patients (diagnosis confirmed by endoscopic Mayo score ≥2) and 26 age-/sex-matched healthy volunteers. Exclusion criteria for all participants: [1] other immune-mediated diseases; [2] malignancy history. Serum was separated by centrifugation and analyzed following the standard protocol provided in the enzyme-linked immunosorbent assay (ELISA) kit. The procedure involved coating a 96-well plate with capture antibodies, adding serum samples for incubation, followed by enzyme-labeled secondary antibodies, and developing the colorimetric reaction. Absorbance was measured using a microplate reader, and the protein concentrations of DLL1, TGFBR2, CCT5, BCR, and DCTN1 were calculated based on the standard curve. Ratios of DLL1/TGFBR2, CCT5/DCTN1, and BCR/DCTN1 were then determined.

### Statistical analysis

The Inverse Variance Weighting (IVW) method was used to evaluate causal relationships, with sensitivity analyses (MR Egger, weighted median, and weighted mode and leave-one-out) performed to ensure robustness. All statistical analyses were conducted using the TwoSampleMR package in R, with significance assessed via two-tailed tests.

Single-cell data analysis was conducted using the Seurat package (v4.3.0), including quality control, dimensionality reduction, and clustering analysis. GO and KEGG enrichment analyses were performed to validate and explore the Mendelian randomization findings.

### Ethics approval

This study was approved by the Institutional Review Committee of the Second Xiangya Hospital, Central South University (Ethics Approval Number:Z0379-01). All participants provided written informed consent prior to the collection of tissue samples. The study was conducted in compliance with the Declaration of Helsinki and relevant national regulations for research involving human subjects.

## Results

### Associations between plasma protein ratios and ulcerative colitis

**Figure 2:**
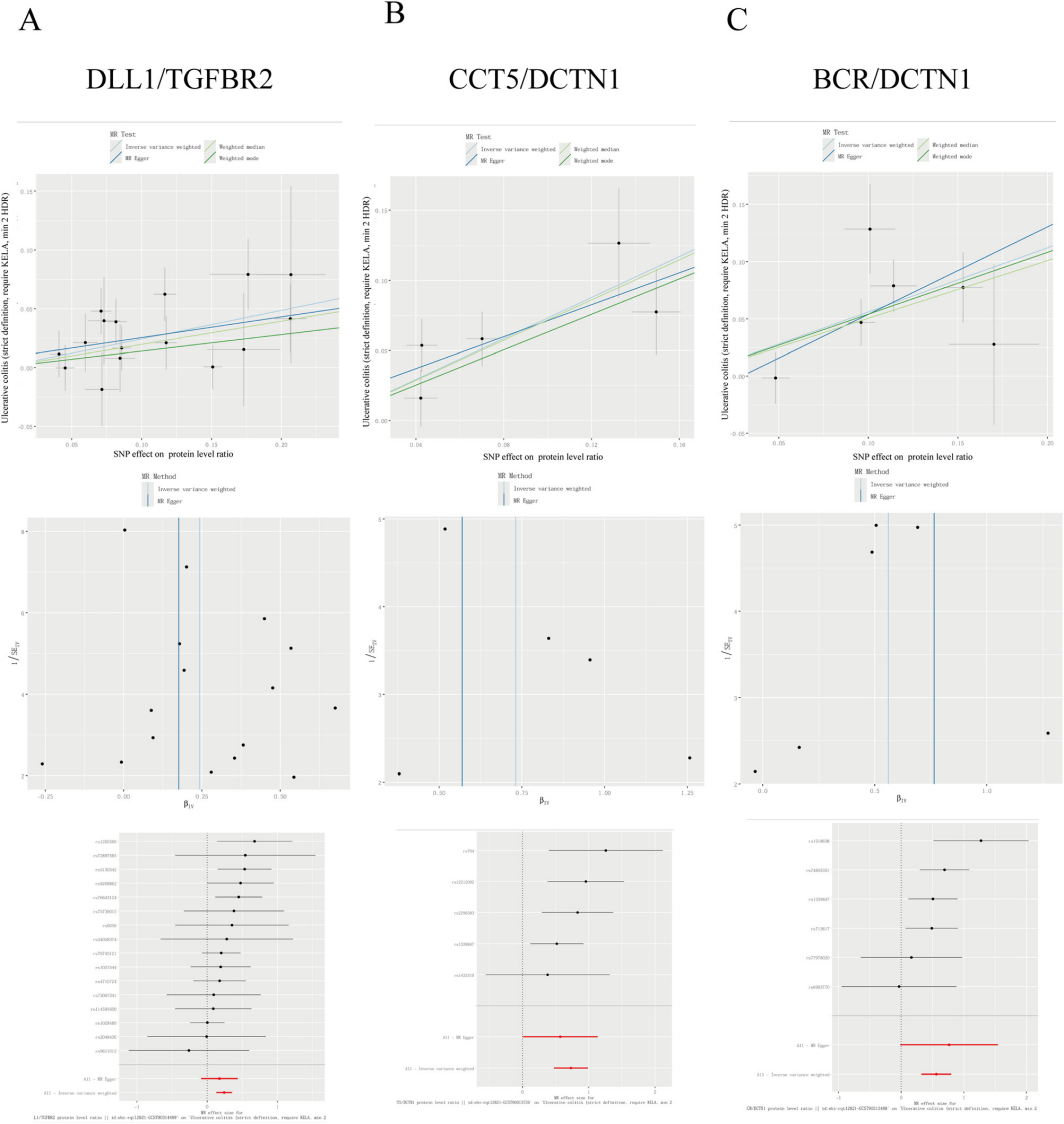
Results of Mendelian randomization analysis. The figure presents Mendelian randomization (MR) analyses for three protein level ratio pairs: (A) DLL1/TGFBR2, (B) CCT5/DCTN1, and (C) BCR/DCTN1. For each pair, the top row shows MR scatter plots, where the x-axis denotes the effect of single nucleotide polymorphisms (SNPs) on the respective protein level ratio, and the y-axis represents the SNP effect on the outcome risk. The middle row displays funnel plots, with the x-axis as the SNP effect on the protein level ratio and the y-axis as precision (inverse standard error) or effect size variance, used to assess heterogeneity or potential bias. The bottom row shows leave-one-out validation forest plots, depicting MR effect estimates (and 95 % CIs) after excluding one SNP at a time to evaluate the robustness of causal inferences.

Using proteomics data from 54,000 samples in the UK Biobank, the association between 2,821 plasma protein ratios and the risk of UC was analyzed. The inverse variance weighted (IVW) method identified 165 protein ratios significantly associated with UC risk (Table S2). Genetically predicted plasma protein ratios demonstrated a range of associations with UC risk per standard deviation increase, from a risk ratio of 0.539 (95 % CI: 0.408–0.712) for the DCTN1/IKBKG ratio to 2.078 (95 % CI: 1.607–2.686) for the CCT5/DCTN1 ratio. Among them, 23 protein ratio pairs with risk ratios exceeding 1.25. Heterogeneity tests for all protein ratios yielded q-values greater than 0.05, indicating no significant heterogeneity among the instrumental variables, thereby enhancing the robustness of the results. Sensitivity analyses using IVW (multiplicative random effects), weighted median, weighted mode, and MR Egger methods demonstrated consistency with the IVW results (Figure 2, Table S3).

### Validation via single-cell transcriptomics

**Figure 3:**
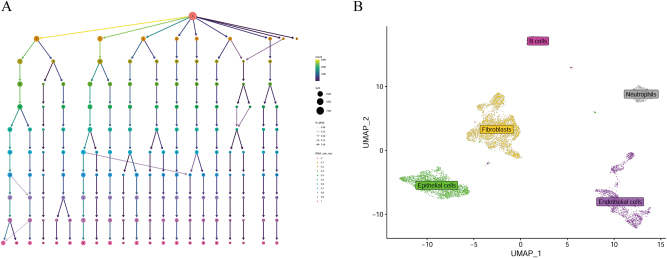
Identification of cell types in single-cell data. (A) Hierarchical clustering tree illustrating relationships among cell subclusters, with each branch representing distinct cell types identified based on molecular markers. (B) UMAP plot showing the distribution of cell clusters. Nine major subclusters were identified, including fibroblasts, epithelial cells, endothelial cells, T cells, neutrophils, and B cells. Cell types are color-coded and labeled for clarity.

To further validate the association between protein level ratios and ulcerative colitis (UC), single-cell transcriptomic data were retrieved from the GEO database, comprising samples from two healthy individuals and two UC patients. Following preprocessing, cells were subjected to dimensionality reduction and clustering based on original molecular markers, resulting in nine subclusters, including fibroblasts, epithelial cells, endothelial cells, T cells, neutrophils, and B cells (Figure 3).

**Figure 4:**
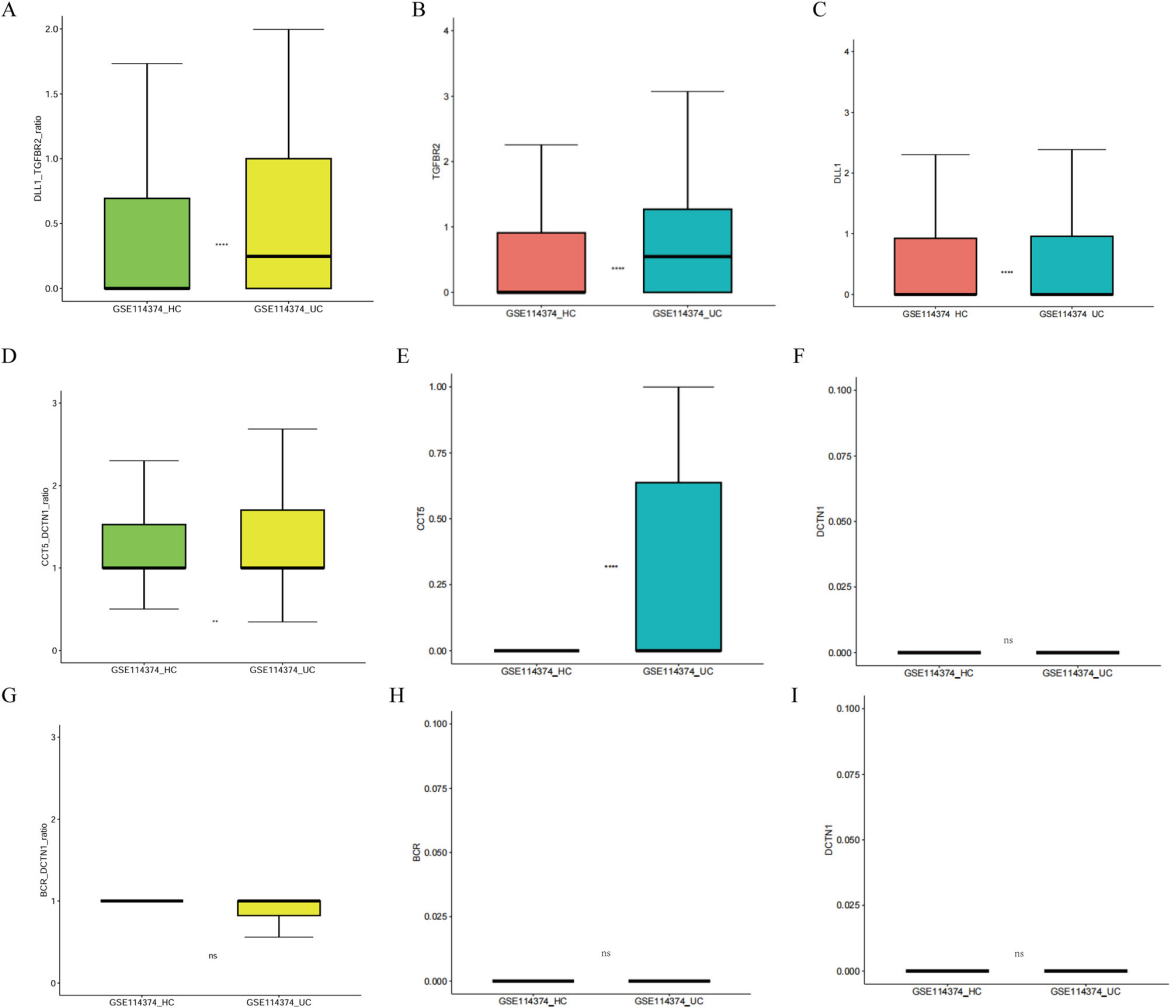
Differential expression levels and ratios of DLL1/TGFBR2, CCT5/DCTN1, and BCR/DCTN1 proteins. (A) Expression differences of DLL1/TGFBR2 between UC patients and healthy controls; (B, C) Differential expression of TGFBR2 and DLL1 proteins, respectively; (D, E) Expression differences of CCT5/DCTN1 ratios and individual proteins (CCT5, DCTN1) between UC and healthy individuals; (G) Differential expression of BCR/DCTN1 ratios; (H, I) Differential expression of BCR and DCTN1 proteins, respectively, between UC patients and healthy controls (HC). ****p<0.0001;**p<0.01;ns:no significance.

The protein levels and ratios of DLL1/TGFBR2, CCT5/DCTN1, and BCR/DCTN1 were visualized across different groups and cell types. The results indicated that DLL1-TGFBR2 protein levels and ratios were significantly higher in UC patients compared to healthy controls. Similarly, the CCT5/DCTN1 protein ratio was elevated in UC patients. Additionally, CCT protein expression levels were markedly higher in UC patients, whereas no significant differences were observed for CCTN1 and BCR/DCTN1 levels (Figure 4).

### Expression of DLL1 and TGFBR2 across cell types

**Figure 5:**
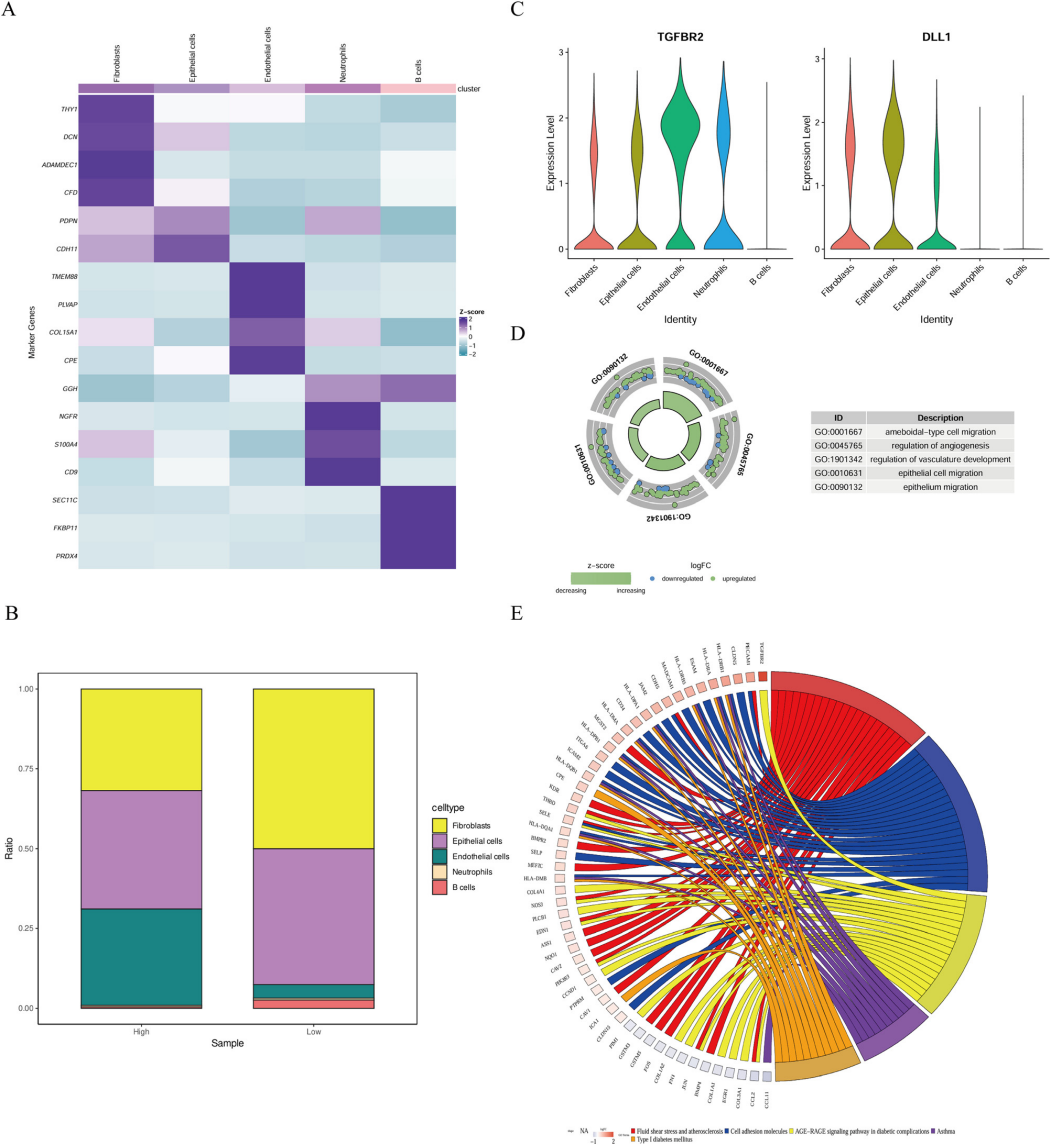
Intercellular distribution of DLL1 and TGFBR2 and results of single-cell pathway enrichment analysis. (A) Cell types and marker genes; (B) Differences in cell types between high and low DLL1/TGFBR2 ratio groups; (C) Intercellular distribution of DLL1 and TGFBR2; (D, E) GO and KEGG pathway enrichment results.

The differential expression of DLL1 and TGFBR2 among cell types was further analyzed. Results revealed that both DLL1 and TGFBR2 were expressed in fibroblasts, epithelial cells, endothelial cells, neutrophils, and B cells. Notably, TGFBR2 exhibited the highest expression in endothelial cells, while DLL1 was predominantly expressed in epithelial and endothelial cells, with moderate expression in fibroblasts and neutrophils and low expression in B cells (Figure 5A). Differential expression analysis was conducted based on cell classification and DLL1/TGFBR2 protein ratio grouping. Enrichment analysis of differentially expressed genes identified significantly enriched pathways. Compared to the low-ratio group, the high-ratio group exhibited a significantly higher proportion of endothelial cells, with elevated expression of genes such as TMEM88 (Transmembrane Protein 88), PLVAP (Plasmalemma Vesicle-Associated Protein), CPE (Carboxypeptidase E), and COL15A1 (Collagen Type XV, Alpha 1) (Figure 5B and C). Gene Ontology (GO) analysis identified 1,002 significantly enriched pathways. The top five pathways included ameboidal-type cell migration, regulation of angiogenesis, regulation of vasculature development, and epithelial cell migration (Figure 5D). Kyoto Encyclopedia of Genes and Genomes (KEGG) analysis identified 98 enriched pathways. Visualization of the top five pathways revealed associations with fluid shear stress and atherosclerosis, cell adhesion molecules (CAMs), the AGE-RAGE signaling pathway in diabetic complications, asthma, and type 1 diabetes mellitus. Associated genes included TGFBR2, PECAM1, CLDN5, ESAM, MADCAM1, CDH5, JAM2, etc (Figure 5E). These results indicate that DLL1 and TGFBR2 proteins are predominantly expressed in endothelial cells and are associated with multiple biological processes and signaling pathways, particularly in angiogenesis and inflammatory responses. The elevated DLL1/TGFBR2 ratio in UC is primarily driven by increased DLL1 expression in epithelial cells and stable TGFBR2 levels in endothelial cells, with endothelial cells emerging as the key cellular compartment mediating this ratio’s association with UC. TGFBR2 may regulate key pathways involved in vascular permeability and repair, such as fluid shear stress and atherosclerosis, cell adhesion molecules (CAMs), and the AGE-RAGE signaling pathway, playing a critical role in UC pathogenesis. Activation or inhibition of these pathways may influence inflammatory cell migration, vascular barrier integrity, and the stability of the intestinal microenvironment in UC.

### Validation of DLL1/TGFBR2, CCT5/DCTN1, and BCR/DCTN1 expression in clinical samples

**Figure 6:**
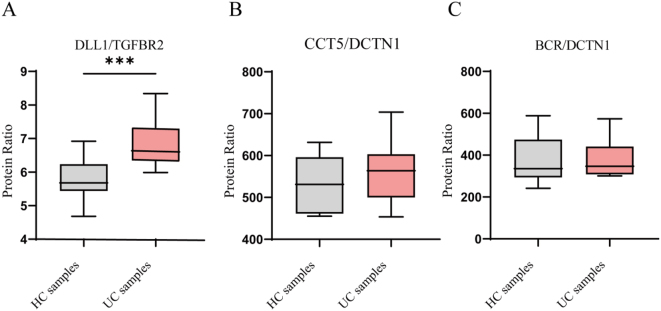
Validation of DLL1/TGFBR2, CCT5/DCTN1, and BCR/DCTN1 expression levels and ratios in clinical samples. (A) The protein ratio of DLL1/TGFBR2 in UC patients and healthy controls (HCs) was compared using ELISA. (B) The protein ratio of CCT5/DCTN1 in UC patients and HC patients was compared using ELISA. (C) The protein ratio of BCR/DCTN1 in UC patients and HC patients was compared using ELISA. ***p<0.001.

Based on single-cell sequencing results – which initially suggested differential expression of DLL1, TGFBR2, CCT5, BCR, and DCTN1, and their derived ratios, in UC – we designed ELISA assays to validate these observations. Blood samples were collected from UC patients, with blood samples from healthy volunteers serving as healthy controls. Proteins were extracted, and the expression levels of DLL1, TGFBR2, CCT5, BCR, and DCTN1 were measured using ELISA across the different groups. Ratios of DLL1/TGFBR2, CCT5/DCTN1, and BCR/DCTN1 were calculated. The results indicated that the DLL1/TGFBR2 ratio was significantly elevated in the UC patient group (Figure 6A). However, the ratios of CCT5/DCTN1 and BCR/DCTN1 did not show statistical differences between the UC patient group and the HC group (Figure 6B and C).

## Discussion

In this study, we utilized proteomic data and SNPs to evaluate the association between 2,821 plasma protein ratios and the risk of UC using MR analysis. A total of 165 significant plasma protein ratios were identified. To ensure the robustness of our results, multiple sensitivity analyses were conducted. To further validate their potential role in UC, we performed single-cell RNA sequencing (scRNA-seq) analysis. The results showed that DLL1/TGFBR2 were highly expressed in endothelial cells, with significant differences in protein ratio levels between UC patients and healthy controls. Enrichment analysis of endothelial cells in UC patients revealed upregulation of pathway proteins associated with vascular integrity and permeability, such as PLVAP. And the enrichment results also indicated that the TGFBR2 gene is associated with the AGE-RAGE signaling pathway. To further confirm the clinical significance of the DLL1/TGFBR2 ratio, we collected blood samples from UC patients and healthy volunteers. The ratios of DLL1/TGFBR2, CCT5/DCTN1, and BCR/DCTN1 were measured using ELISA, providing additional evidence supporting the clinical value of the DLL1/TGFBR2 ratio.

Previous studies have demonstrated that TGF-β (transforming growth factor-β) exerts complex roles in UC, encompassing anti-inflammatory and immunoregulatory functions, as well as involvement in tissue repair and fibrosis processes [11], [12], [13], [14]. TGF-βmaintains immune tolerance and suppresses excessive inflammatory responses by inhibiting the production of pro-inflammatory cytokines and promoting the differentiation of regulatory T cells (Tregs). Additionally, TGF-β promotes epithelial cell proliferation and migration, facilitating intestinal wound healing and maintaining barrier integrity. However, sustained activation of the TGF-β signaling pathway may lead to intestinal fibrosis and tumor development, indicating its complex role in UC as both protective and disease-promoting [15]. TGFBR2, as a key component of the TGF-β signaling pathway, plays a critical role in regulating immune responses and suppressing inflammation [16], 17]. DLL1 (Delta-like 1) is an important transmembrane protein and a ligand of the Notch signaling pathway. DLL1 activates the Notch signaling pathway by binding to Notch receptors, thereby regulating immune cell differentiation and function [18], [19], [20].

The AGE-RAGE signaling pathway is a complex signaling network driven by the interaction between advanced glycation end products (AGEs) and their receptor RAGE (Receptor for AGEs). AGEs are irreversible products generated by non-enzymatic glycation reactions between glucose or lipids and proteins, and their accumulation is closely associated with oxidative stress [21]. In diabetes, hyperglycemia significantly accelerates AGEs production, and abnormal activation of RAGE is one of the core pathological mechanisms of diabetic complications. For example, the AGE-RAGE pathway promotes glomerular mesangial cell proliferation, extracellular matrix deposition, and fibrosis by activating TGF-β1 and NF-κB, ultimately leading to glomerulosclerosis and diabetic nephropathy [22]. Although previously reported to be associated with diabetes, this pathway also plays a role in UC, where persistent inflammation increases oxidative stress and promotes AGEs generation [23]. For instance, harmacological analysis has found that Wumeiwan (WMW) can inhibit the production of CXCL1, MCP-1, IL-6, and TNF-α by regulating the RAGE signaling pathway, thereby suppressing angiogenesis and inflammation in UC and alleviating mucosal inflammation [24]. The AGE-RAGE signaling pathway may regulate vascular permeability and apoptosis, affecting intestinal barrier function and inflammatory cell migration, thus playing a pivotal role in UC pathogenesis and progression [24], 25]. Additionally, studies have found that high-fructose diets contribute to the formation, activation, and upregulation of AGEs, leading to increased numbers and activation of mast cells. The interaction between mast cells and goblet cells results in mucus discharge disorders, causing mucosal irritation and intestinal mucus barrier dysfunction in mice [26]. Another study investigated RAGE-dependent sustained activation of NF-κB in resected intestinal specimens from patients with inflammatory bowel disease [27]. Therefore, targeting the AGE-RAGE signaling pathway may offer novel therapeutic strategies for the treatment of UC.

Notably, the inconsistent ELISA results for CCT5/DCTN1 and BCR/DCTN1 compared to MR findings warrant discussion. First, the MR analysis relies on genetically predicted plasma protein ratios using SNPs as instruments, reflecting transcriptional regulation, while ELISA measures total protein abundance in serum, which may be influenced by post-translational modifications that alter protein function or interactions without changing overall expression levels. Second, the MR data are derived from European populations (UK Biobank, FinnGen), whereas the ELISA samples include Chinese UC patients and healthy controls, potentially introducing ethnic or environmental heterogeneity that impacts genetic-protein associations. Additionally, the ELISA sample size (26 pairs) is relatively small, limiting statistical power to detect modest effects, and MR’s reliance on summary-level GWAS data precludes stratified analyses of individual-level factors (e.g., disease activity, medication use) that might influence protein ratios.

Notably, the application of artificial intelligence in structural biology, such as the AlphaFold model, offers transformative opportunities to address the unresolved protein interaction mechanisms in this study [28]. AlphaFold’s ability to predict 3D structures and binding interfaces of proteins (e.g., DLL1 and TGFBR2) could elucidate how their ratio dysregulation contributes to UC pathogenesis. For instance, identifying key binding domains of TGFBR2 involved in angiogenesis or inflammatory signaling (e.g., through predicted complex structures) would provide mechanistic insights into its role in vascular permeability. Additionally, AlphaFold-generated structures of DLL1 isoforms could guide virtual screening for small-molecule inhibitors targeting the DLL1/TGFBR2 axis, potentially accelerating therapeutic development for UC.

This study has several methodological strengths. First, the genetic data on plasma protein ratios and UC were derived from a well-designed, large-scale genome-wide association study (GWAS), minimizing the risk of confounding effects caused by sample overlap. Second, all selected genetic instruments had F-statistics greater than 10, indicating strong instrument strength, and we caution that this does not fully preclude weak instrument bias in ratio-based MR analyses. Finally, the integration of multiple sensitivity analysis techniques further enhanced the robustness of our findings.

Three fundamental limitations warrant emphasis. First, the inherent limitations of Mendelian randomization, including phenotypic heterogeneity and developmental compensation, may compromise the precision and applicability of our results. Second, our reliance on summary-level data limited our ability to perform stratified analyses or explore individual-level data in depth. Third, as our study population was primarily of European ancestry, limiting the generalizability of our findings to non-European populations where genetic architecture and disease epidemiology may differ. Replication in diverse cohorts is essential to validate the robustness of these associations across ethnic groups. Finally, although MR suggests a causal relationship between plasma protein ratios and UC, the underlying molecular mechanisms – such as specific signaling pathways or cell types involved – remain unclear.

In conclusion, this study validated a causal relationship between DLL1/TGFBR2 protein ratios and the occurrence of UC through Mendelian randomization and single-cell sequencing analyses. UC patients should consider timely therapeutic interventions to halt disease progression. For patients with Crohn’s disease, targeting DLL1/TGFBR2 may inhibit TNFα and NLR signaling pathways, potentially leading to improved therapeutic outcomes. In summary, these findings provide novel insights and potential strategies for the diagnosis and treatment of ulcerative colitis.

## Supplementary Material

Supplementary Material
